# Adipocytes and Stromal Cells Regulate Brown Adipogenesis Through Secretory Factors During the Postnatal White-to-Brown Conversion of Adipose Tissue in Syrian Hamsters

**DOI:** 10.3389/fcell.2021.698692

**Published:** 2021-07-05

**Authors:** Junnosuke Mae, Kazuki Nagaya, Yuko Okamatsu-Ogura, Ayumi Tsubota, Shinya Matsuoka, Junko Nio-Kobayashi, Kazuhiro Kimura

**Affiliations:** ^1^Laboratory of Biochemistry, Faculty of Veterinary Medicine, Hokkaido University, Sapporo, Japan; ^2^Laboratory of Histology and Cytology, Faculty of Medicine and Graduate School of Medicine, Hokkaido University, Sapporo, Japan

**Keywords:** adipocyte, brown adipose tissue(BAT), uncoupling protein 1(UCP1), Syrian hamster, secretory factor

## Abstract

Brown adipose tissue (BAT) is a specialized tissue that regulates non-shivering thermogenesis. In Syrian hamsters, interscapular adipose tissue is composed primarily of white adipocytes at birth, which is converted to BAT through the proliferation and differentiation of brown adipocyte progenitors and the simultaneous disappearance of white adipocytes. In this study, we investigated the regulatory mechanism of brown adipogenesis during postnatal BAT formation in hamsters. Interscapular adipose tissue of a 10-day-old hamster, which primarily consists of brown adipocyte progenitors and white adipocytes, was digested with collagenase and fractioned into stromal–vascular (SV) cells and white adipocytes. SV cells spontaneously differentiated into brown adipocytes that contained multilocular lipid droplets and expressed uncoupling protein 1 (Ucp1), a marker of brown adipocytes, without treatment of adipogenic cocktail such as dexamethasone and insulin. The spontaneous differentiation of SV cells was suppressed by co-culture with adipocytes or by the addition of white adipocyte-conditioned medium. Conversely, the addition of SV cell-conditioned medium increased the expression of *Ucp1*. These results indicate that adipocytes secrete factors that suppress brown adipogenesis, whereas SV cells secrete factors that promote brown adipogenesis. Transcriptome analysis was conducted; however, no candidate suppressing factors secreted from adipocytes were identified. In contrast, 19 genes that encode secretory factors, including bone morphogenetic protein (BMP) family members, BMP3B, BMP5, and BMP7, were highly expressed in SV cells compared with adipocytes. Furthermore, the SMAD and MAPK signaling pathways, which represent the major BMP signaling pathways, were activated in SV cells, suggesting that BMPs secreted from SV cells induce brown adipogenesis in an autocrine manner through the SMAD/MAPK signaling pathways. Treatment of 5-day-old hamsters with type I BMP receptor inhibitor, LDN-193189, for 5 days reduced p38 MAPK phosphorylation and drastically suppressed BAT formation of interscapular adipose tissue. In conclusion, adipocytes and stromal cells regulate brown adipogenesis through secretory factors during the postnatal white-to-brown conversion of adipose tissue in Syrian hamsters.

## Introduction

Mammals have two types of adipose tissue: white adipose tissue (WAT) and brown adipose tissue (BAT). WAT stores energy as triglyceride, and white adipocytes contain a large unilocular lipid droplet and small cytoplasm. In contrast, BAT is specialized for non-shivering thermogenesis and dissipates energy as heat through the activation of mitochondrial uncoupling protein 1 (UCP1) (Cannon and Nedergaard, 2004). Brown adipocytes contain multilocular lipid droplets and abundant mitochondria. The physiological role and morphology of WAT and BAT are significantly different; however, they may convert their appearance into one another under specific physiological conditions (Lee et al., 2014). Brown-like adipocytes, known as beige/brite adipocytes, which contain multilocular lipid droplets and express UCP1, are induced in WAT during chronic sympathetic stimulation such as cold exposure (Cousin et al., 1992; Jimenez et al., 2003; Contreras et al., 2014). This change is referred to as “browning of WAT.” In contrast, obesity-induced “whitening of BAT” occurs when the lipid content increases from overnutrition and the lipid droplets change to a unilocular form, similar to that in white adipocytes (Sellayah and Sikder, 2014; Shimizu et al., 2014). Thus, adipose tissues undergo drastic remodeling by environmental stimuli, and the underlying mechanisms, especially the role of local environment, for these changes have not been fully elucidated.

Syrian hamsters are a unique model to investigate the mechanism of adipose tissue remodeling. In most mammals, BAT development is completed before or soon after birth because BAT thermogenesis is indispensable in neonates and infants. In contrast, Syrian hamsters are born without BAT as it develops postnatally by the time of weaning (Smalley and Smalley, 1967; Houstek et al., 1990) when the complete homeothermic phenotype is acquired (Tsubota et al., 2019). Interscapular adipose tissue, where BAT is present in other animals, is occupied by white adipocytes at birth. Brown adipocyte progenitors appear at the edge of the tissue, proliferate and increase in number, and then differentiate into brown adipocytes (Okamatsu-Ogura et al., 2018). This white-to-brown conversion of adipose tissue is also accompanied by active vascularization along with the proliferation of vascular endothelial cells (Okamatsu-Ogura et al., 2018).

It is unclear how this adipose tissue conversion is regulated; however, environmental temperature appears to have a distinct role because the proliferation of the progenitor and endothelial cells is suppressed in pups raised in a warm environment (Nagaya et al., 2019). However, the BAT formation process itself occurs and functional BAT is formed even in a warm environment, suggesting that there may be some cell-intrinsic regulatory mechanism. Interestingly, the process of BAT formation in hamsters is also the process in which white adipocytes gradually disappear. White adipocytes not only store excess energy as triglycerides, but also secrete various adipokines that regulate the metabolism of other tissues, not only locally, but systemically. Some of the adipokines modulate adipose tissue remodeling in an autocrine or paracrine manner (Goralski et al., 2007; Than et al., 2012; Choi et al., 2020). In addition, certain populations of stromal cells have been reported to modulate adipogenesis in a paracrine manner (Ruan et al., 2002; Sakaue et al., 2002; Hutley et al., 2004; Goto et al., 2016; Meissburger et al., 2016; Machida et al., 2018; Schwalie et al., 2018; Buffolo et al., 2019). Therefore, it is possible that brown adipogenesis is regulated by surrounding stromal cells or white adipocytes through secretory factors (Poulos et al., 2010). In this study, we evaluated the regulatory mechanism of brown adipogenesis during postnatal white-to-brown conversion of adipose tissue in hamsters.

## Materials and Methods

### Animals

The experimental procedures and care of animals were approved by the Animal Care and Use Committee of Hokkaido University (Sapporo, Japan) and conducted in an animal facility approved by the Association for Assessment and Accreditation of Laboratory Animal Care International. Parental Syrian hamsters were purchased from Japan SLC (Hamamatsu, Japan) and housed in plastic cages in an air-conditioned room at 23°C along with a 12:12 h light:dark cycle. They were given free access to laboratory chow (Oriental Yeast, Tokyo, Japan) and tap water. Male and female hamsters were mated by putting a female into a cage with a male for 1 week. At the indicated day, pups or adult hamsters were euthanized by intraperitoneal injection of pentobarbital (Somnopentyl; Kyoritsu Seiyaku Co., Tokyo, Japan). In experiment of acute effect of BMP type I receptor inhibitor LDN-193189 (LDN, 3 mg/kg; R & D System, Minneapolis, MN, United States), 10-day-old hamsters were subcutaneously injected with LDN or vehicle (0.5 w/v% carboxymethylcellulose sodium, 0.9 w/v% NaCl, 0.4 v/v% Tween80, 0.9 v/v% benzyl alcohol), and euthanized by intraperitoneal injection of pentobarbital 30 min later. In experiment of chronic effect of LDN, 5-day-old hamsters were subcutaneously injected with LDN (3 mg/kg) or vehicle once a day for 5 days and euthanized by intraperitoneal injection of pentobarbital 24 h after the last injection. The interscapular and inguinal adipose tissues were collected.

### Histological Analysis

Tissue specimens were fixed in 10%-buffered formalin and embedded in paraffin according to the conventional method, cut into 4-μm-thick sections, and stained with hematoxylin and eosin.

### Isolation of Adipocytes and Stromal–Vascular (SV) Cells

Tissues were minced into small pieces and incubated with DMEM-high glucose (FUJIFILM Wako Pure Chemicals Corporation, Osaka, Japan) containing 1% fatty acid-free BSA (FUJIFILM Wako Pure Chemicals Corporation) and 2 mg/ml collagenase (FUJIFILM M Wako Pure Chemicals Corporation) at 37°C for 1 h while shaking at 90 cycles/min. The suspension was filtered through a 200-μm nylon filter and centrifuged at room temperature at 120 *g* for 5 min. The floating cells were collected as the mature adipocyte fraction. The pellet was re-suspended in a hemolytic buffer (150 mM NH_4_Cl, 10 mM KHCO_3_, 0.1 mM Na_2_EDTA, pH 7.4), and passed through a 25-μm nylon filter. The filtrate was then centrifuged at 120 *g* for 5 min, and the pellet obtained represented the SV cells.

### Preparation of Conditioned Medium

Adipocyte fractions derived from the interscapular or inguinal adipose tissues of three pups were pooled and cultured in an OptiCell cell-culture system (BioCrystal, Ohio State, United States). This enables culture of the adipocytes floating in the upper layer of the medium while protecting them from drying out by using gas-permeable membranes with efficient O_2_ and CO_2_ exchange. The OptiCell chamber was filled with 10 ml of culture medium [10% fetal calf serum (Cytiva, Tokyo, Japan), 100 U/ml penicillin, 100 μg/ml streptomycin-containing DMEM-high glucose] and cells were cultured at 37°C and 5% CO_2_. SV cells derived from the interscapular adipose tissues of three pups were cultured in 35-mm dishes coated with Type I collagen (IWAKI AGC Techno Glass Co., Ltd., Shizuoka, Japan) at a density of 7.5 × 10^5^ cells/dish. 3 days later, the culture medium was centrifuged at 200 × *g* for 5 min to remove the cells and filtered through a 0.2-μm filter. The filtrate was obtained as conditioned medium and stored at –20°C.

### Primary Culture

Stromal–vascular cells derived from the interscapular or inguinal adipose tissues were cultured in a 35-mm dish coated with Type I collagen (IWAKI AGC Techno Glass Co., Ltd.) at a density of 7.5 × 10^5^ cells/dish in culture media with or without conditioned medium. In co-culture experiments, the SV cell fractions obtained from the three pups were suspended in 10-ml culture medium as one pool and cultured in the OptiCell chamber (BioCrystal) for 3 days with or without the pooled adipocyte fraction obtained from the three pups.

### Real-Time PCR

Cells were treated with RNAiso (Takara Bio, Shiga, Japan), and total RNA was extracted according to the manufacturer’s instructions. Total RNA was reverse-transcribed using a 15-mer oligo(dT) adaptor primer and M-MLV reverse transcriptase (Thermo Fisher Scientific, Waltham, MA, United States). Real-time PCR was performed using a fluorescence thermal cycler (LightCycler system, Roche Diagnostics, Mannheim, Germany) and FastStart Essential DNA Green Master Mix (Roche Diagnostics). Absolute expression levels were determined using a standard curve using respective cDNA fragments as standards. The mRNA levels are expressed as relative values compared with *Actb* mRNA levels. The primers used in this study are listed in Table 1.

**TABLE 1:** Primers for real-time PCR.

Gene name (Gene symbol)	Primer sequences
*Actin beta (Actb)*	Forward: 5′-AAG TGT GAC GTT GAC ATC CCG-3′
	Reverse: 5′-GAT CCA CAC AGA GTA CTT GC-3′
*Bone morphogenetic protein 3b (Bmp3b)*	Forward: 5′-CGGACATCGGGTGGAATGAA -3′
	Reverse: 5′-TCTGGAACACAACAGGGCTC -3′
*Bone morphogenetic protein 5 (Bmp5)*	Forward: 5′-TCTCATCAGGACCCCTCCAG-3′
	Reverse: 5′-CCCTTCCGGTGCTATGATCC-3′
*Bone morphogenetic protein 7 (Bmp7)*	Forward: 5′-GGG CTG TAA CTC TGG CAC AT-3′
	Reverse: 5′-ACG TGG CCA ATG GAC TCT TT-3′
*Fatty acid binding protein 4 (Fabp4)*	Forward: 5′-GAG ATG TGA TCA CCA TCC GAA C-3′
	Reverse: 5′-ACC CTC ACG CTT CCT CTT TA-3′
*Fatty acid synthase (Fasn)*	Forward: 5′-AAG TAC CAT GGC AAC GTG AC-3′
	Reverse: 5′-CAA TGA TGT GCA CAG ACA CC-3′
*Hormone sensitive lipase (Hsl)*	Forward: 5′-CTC TAA GTG TGT CAG TGC CT-3′
	Reverse: 5′-TCC AGG AAG GAG TTG AGC CA-3′
*Leptin (Lep)*	Forward: 5′-TCT GCA GGA CAT TCT CCG AC-3′
	Reverse: 5′-AGA CAC CTG GAA GCT AAG GC-3′
*Lipoprotein lipase (Lpl)*	Forward: 5′-TCT CTT CAT TGA CTC CCT GCT G-3′
	Reverse: 5′-GTC TTC AGG TAC ATC TTG CTG C-3′
*Peroxisome proliferator activated receptor gamma (Pparg)*	Forward: 5′-TTA GAT GAC AGT GAC TTG GC-3′
	Reverse: 5′-CTC GAT GGG CTT CAC GTT CA-3′
*Uncoupling protein 1 (Ucp1)*	Forward: 5′-GAG CTG GTA ACA TAT GAC CT -3′
	Reverse: 5′- TGT CCT GGC AGA GAG TTG AT -3′

### Next-Generation Sequence Analysis (mRNA-Seq Analysis)

Total RNA (5 μg) extracted from the SV cells or adipocyte fractions derived from the interscapular and inguinal adipose tissue was prepared. Next-generation sequence analysis was performed by Eurofin Genomics Co., Ltd. (Tokyo, Japan) using a HiSeq 2500 system (Illumina, San Diego, CA, United States). Ingenuity Pathway Analysis software (Qiagen, Hilden, Germany) was used for GO analysis. The set of genes, in which the ratio of expression in interscapular SV cells relative to interscapular adipocytes was greater than 5, was extracted, and enrichment analysis was conducted using Metascape^1^. For enriched GO, p-values were less than 0.01, the enrichment factor was greater than 1.5, and the “signaling (GO:0023052)” as Parent GO was extracted.

### Western Blotting

Tissue specimens were homogenized in ice-cold Tris-ethylenediaminetetraacetic acid (EDTA) buffer (10 mM Tris and 1 mM EDTA, pH 7.4) containing cocktails of phosphatase inhibitor (Nacalai Tesque, Kyoto, Japan) and protease inhibitor (Sigma-Aldrich, St. Louis, MO, United States). After centrifugation at 800 × *g* for 20 min at 4°C, the resulting supernatant obtained as total protein was used for western blotting analysis. In brief, the protein was separated by SDS-PAGE and transferred to a polyvinylidine fluoride membrane (Immobilon; Millipore, Tokyo, Japan). After blocking with 5% skimmed milk (Morinaga Milk Industry Co., Tokyo, Japan) or 2% BSA, the membrane was incubated with a primary antibody overnight. Primary antibodies against phospho-SMAD1,5 (catalog number #9516), total SMAD1 (#6944), phospho-p44,p42 mitogen-activated protein kinase (MAPK: ERK; #9101), total ERK (#9102), phospho-p38 MAPK (#4511), total p38 MAPK (#9212) were purchased from Cell Signaling Technology (Beverly, MA, United States), and for Tubulin (#T5168) from Sigma-Aldrich. The bound antibody was visualized using an enhanced chemiluminescence system (GE Healthcare UK Ltd., Little Chalfont, Bucks, United Kingdom) using a horseradish peroxidase-linked secondary antibody.

### Data Analysis

Values are expressed as the mean ± standard error. Statistical analyses were performed using a Student’s *t*-test or two-way analysis of variance followed by the Tukey–Kramer *post hoc* test.

## Results

**FIGURE 1:**
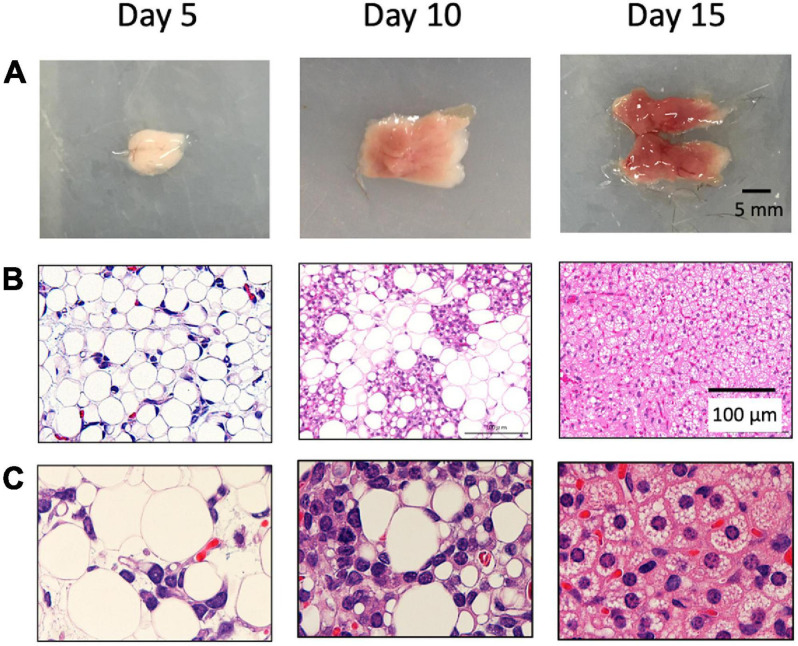
Postnatal development of brown adipose tissue in hamsters. Interscapular adipose tissue was obtained from 5– to 15-day-old Syrian hamsters. Gross images **(A)** and histological images **(B,C)** are shown. In 5-day-old, the tissue was mainly composed of white adipocytes, which disappeared thereafter. In the 10-day-old hamsters, brown adipocyte progenitors were observed and surrounded by white adipocytes. The tissue was primarily composed of brown adipocytes in the 15-day-old hamsters.

First, we histologically analyzed interscapular adipose tissue collected from 5– to 15-day-old hamsters. The color of the tissue was white in the 5-day-old hamster, gradually changed to a brownish color, and showed a typical BAT appearance in the 15-day-old hamster (Figure 1A). Histologically, the tissue of the 5-day-old hamster mainly consisted of white adipocytes containing unilocular lipid droplets, whereas the tissue of the 15-day-old hamster primarily consisted of brown adipocytes with multilocular lipid droplets (Figures 1B,C). In the 10-day-old hamster, the non-adipocyte cells with large nucleus, which suggested a brown adipocyte progenitor in a previous study, presented as a cluster surrounded by white adipocytes.

**FIGURE 2:**
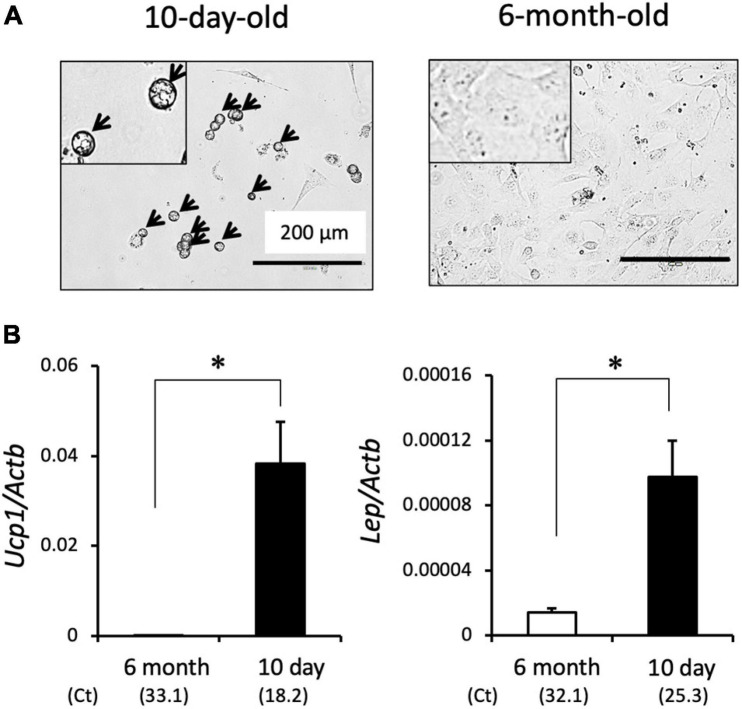
Spontaneous brown adipogenesis of SV cells isolated from interscapular adipose tissue of 10-day-old hamsters. Interscapular adipose tissues collected from 10-day-old or 6-month-old hamsters were digested by collagenase treatment and the stromal–vascular (SV) cell fraction was collected by centrifugation. SV cells were cultured for three days without adipogenic induction cocktail treatment. **(A)** Representative microscopic images of primary cultured SV cells. Cells from 10-day old hamsters showed round shape with multilocular lipid droplets (arrows), whereas those from 6-month-old hamsters showed fibroblast-like shape without lipid droplet. Insets show higher magnification images. **(B)** Expression of the *Ucp1* and *Lep* genes were measured by real-time PCR. The expression values were normalized to the expression of *Actb* and expressed as mean values ± standard error of three and four independent experiments for the 6-month and 10-day group, respectively. An asterisk indicates a significant difference (*p* < 0.05) between groups. The mean Ct values are shown in parentheses.

We previously showed that SV cells isolated from interscapular adipose tissues differentiate into brown adipocytes when treated with adipogenic cocktail such as dexamethasone and insulin (Okamatsu-Ogura et al., 2018). Interestingly, SV cells isolated from the interscapular adipose tissue of a 10-day-old hamster spontaneously differentiated into adipocytes that contain lipid droplets without treatment with adipogenic cocktail (Figure 2A). These cells expressed genes encoding the adipokine, *Lep*, and brown adipocyte marker, *Ucp1*, indicating differentiation into brown adipocytes (Figure 2B). In contrast, SV cells isolated from adult hamsters failed to differentiate into adipocytes, and the expression of *Lep* and *Ucp1* was extremely low under these conditions. These results indicate that brown adipocyte progenitors in SV cells isolated from developing hamsters can spontaneously differentiate into brown adipocytes even without adipogenic cocktail treatment.

**FIGURE 3:**
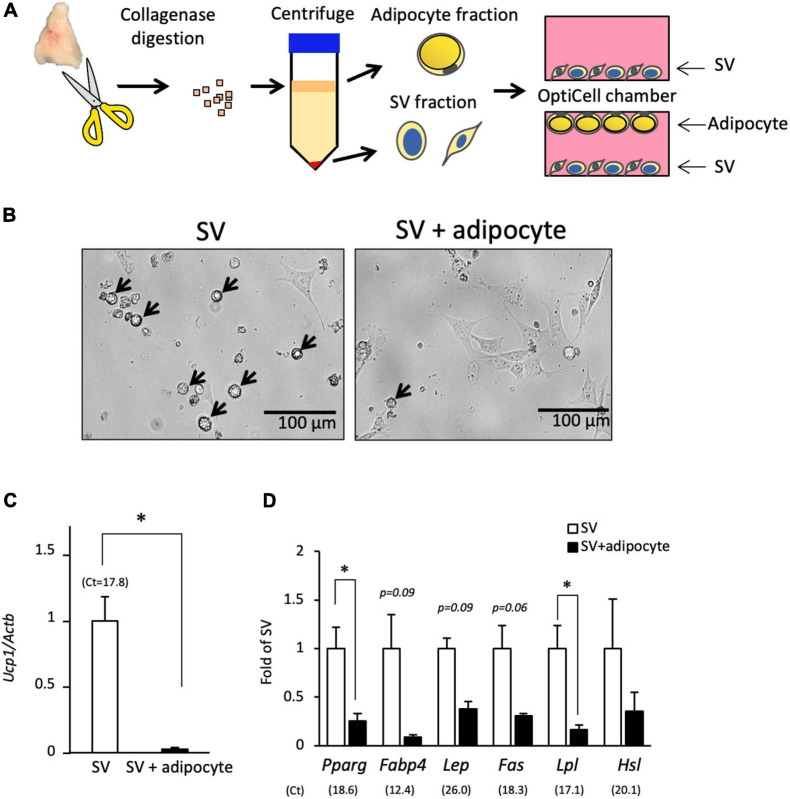
Co-culture with adipocytes suppresses the spontaneous differentiation of SV cells. **(A)** Stromal–vascular (SV) cells and adipocytes were fractionated by centrifugation after collagenase digestion of the interscapular adipose tissue of 10-day-old hamsters. SV cells were cultured in an OptiCell chamber with or without adipocytes for three days without adipogenic induction cocktail treatment. Adipocytes floated at the top of the medium, and SV cells were attached at the bottom of the chamber. **(B)** Representative microscopic image of the bottom side of the OptiCell chamber is shown. SV cells cultured alone showed spontaneous differentiation into lipid droplet-containing adipocytes (arrows), but lipid droplet-containing cells were not observed when SV cells were co-cultured with adipocytes. The expression of *Ucp1***(C)** and marker genes of adipogenesis or mature adipocyte **(D)** in SV cells cultured with or without adipocytes for 3 days were measured by real-time PCR. The expression of each gene was normalized to the expression of *Actb* and expressed relative to the value of the SV cells cultured alone. Values represent means ± standard error for three independent experiments. An asterisk indicates a significant difference (*p* < 0.05) between groups. The mean Ct values in the SV cells cultured alone are shown in parentheses.

Next, we examined the effect of co-culture with adipocytes on the spontaneous differentiation of SV cells (Figure 3A). In culture system using an OptiCell chamber, adipocytes floated to the top, and SV cells attached to the bottom side. When cultured alone, SV cells spontaneously differentiated into lipid droplet-containing adipocytes (Figure 3B), similar to the results shown in Figure 2. However, lipid droplet-containing cells were not observed when SV cells were co-cultured with adipocytes. Expression of *Ucp1* was significantly lower in the co-culture group compared with the single culture group (Figure 3C). The expressions of adipogenesis marker *Pparg* and its target gene *Lpl* were also significantly lower in the co-culture group compared with the single culture group (Figure 3D). The expressions of mature adipocyte markers such as *Fabp4*, *Lep*, *Fas*, and *Hsl* tended to, but not significantly, be lower in the co-culture group compared with the single culture group. These results indicate that co-culture with adipocytes suppresses the spontaneous differentiation of the progenitors into brown adipocytes.

**FIGURE 4:**
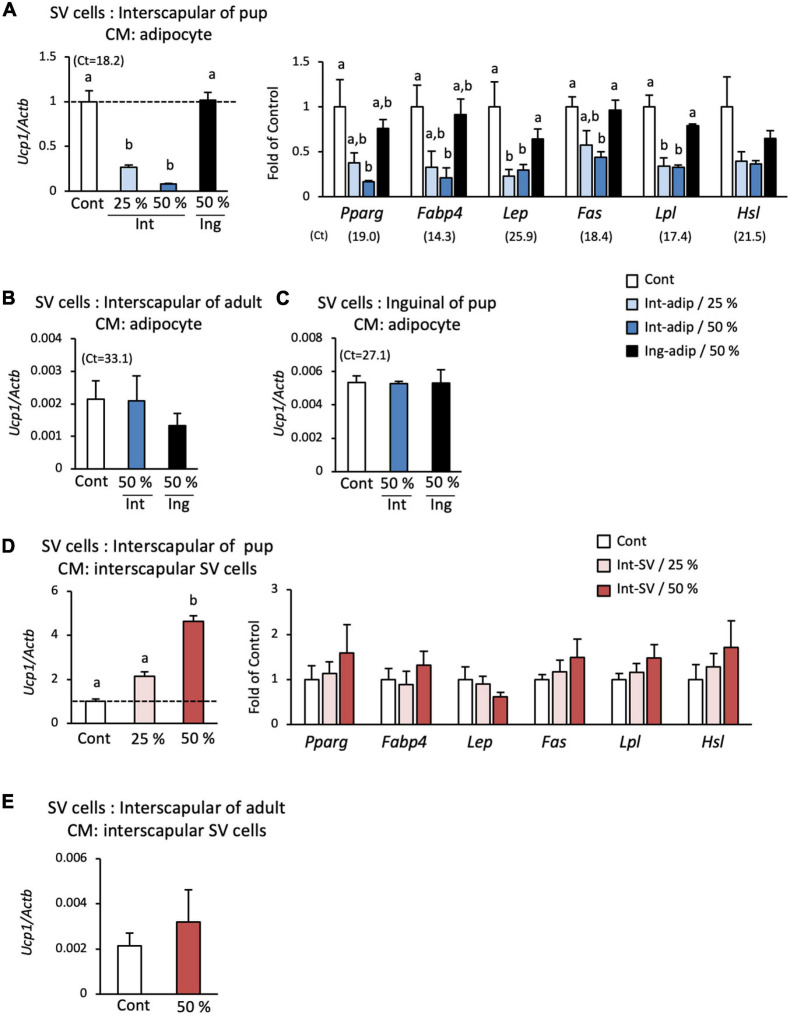
Effect of conditioned medium from adipocytes and SV cells on the spontaneous differentiation of SV cells. Stromal–vascular (SV) cells and adipocytes were fractionated by centrifugation after collagenase digestion of the interscapular (Int) or inguinal (Ing) adipose tissue of 10-day-old hamsters. Adipocytes or SV cells were cultured for three days, and the medium was collected and designated adipocyte- or SV cell-conditioned medium. SV cells from interscapular adipose tissue from 10-day-old hamsters **(A,D)** or 6-month-old hamsters **(B,E)**, and inguinal adipose tissue of 10-day-old hamsters **(C)** were cultured without adipogenic induction cocktail treatment in culture media containing the adipocyte- **(A-C)** or SV cell-conditioned medium **(D,E)** at the indicated ratio. The expressions of *Ucp1* and marker genes of adipogenesis or adipocyte were measured after the 3-day culture. The expression of each gene was normalized to the expression of *Actb* and expressed relative to the value of the control interscapular SV cells from 10-day-old hamsters. Values represent means ± standard error for four independent experiments. Different letters indicate significant differences (*p* < 0.05) between groups. The mean Ct values in the control group are shown in parentheses.

Suppression of the spontaneous differentiation of progenitors by co-culture with adipocytes may result from direct cell–cell interaction or an indirect interaction through secretory factors. To examine this in more detail, a conditioned medium was prepared from interscapular adipocytes, and its effect on differentiation was determined. When SV cells were cultured with a low concentration (25%) of the conditioned medium, the expression of *Ucp1* was significantly reduced to approximately 30% of that in cells cultured without the conditioned medium (Figure 4A). *Ucp1* expression was further reduced when cultured with a high concentration (50%) of the conditioned medium. Conditioned medium from interscapular adipocytes also suppressed the expression of marker genes for adipogenesis or mature adipocyte (Figure 4A). On the other hand, conditioned medium prepared from adipocytes isolated from the inguinal adipose tissue, a traditional WAT depot, showed no effect on *Ucp1* and adipocyte marker gene expressions in SV cells. When SV cells from adult hamsters (Figure 4B) or those from the inguinal adipose tissue of 10-day-old pups (Figure 4C) were used, spontaneous differentiation was not observed and the expression of *Ucp1* was extremely low compared with that in SV cells from the interscapular adipose tissue of 10-day-old hamsters. Effect of interscapular adipocyte-conditioned medium on *Ucp1* expression was not observed. These results indicate that interscapular adipocytes from developing hamsters secrete a factor(s) that suppresses the differentiation of brown adipocyte progenitors.

To examine the possibility that SV cells also secrete factors that regulate brown adipogenesis, the effect of SV cell-conditioned medium was also examined. When interscapular SV cells from 10-day-old hamsters were cultured in a low (25%) or high (50%) concentration of interscapular SV cell-conditioned medium, the expression of *Ucp1* increased in a dose-dependent manner and was more than 2- and 4-fold higher than that in the control group, respectively (Figure 4D). In contrast, conditioned medium from interscapular SV cells showed no effect on the expressions of marker genes for adipogenesis or mature adipocyte (Figure 4D). SV cell-conditioned medium from adult hamsters failed to show any effect on *Ucp1* expression (Figure 4E). These results indicate that SV cells secrete a factor(s) that enhances brown adipogenic program, but not adipogenesis itself, during spontaneous differentiation of progenitors.

**FIGURE 5:**
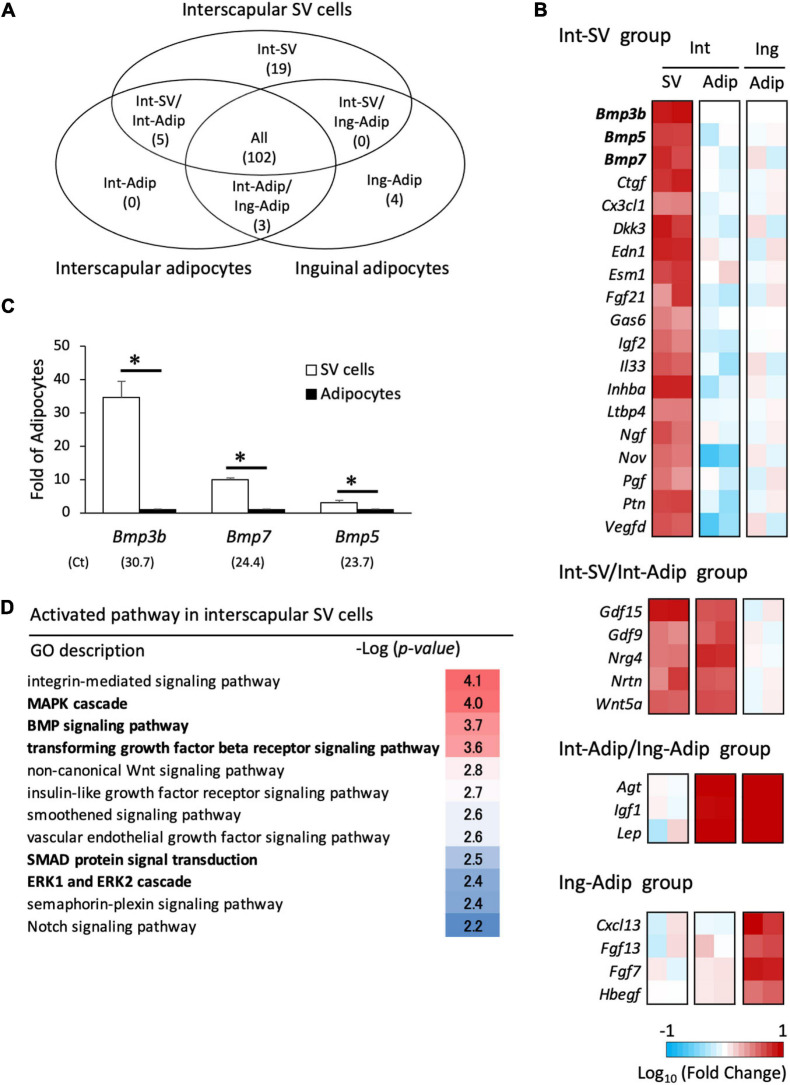
Transcriptome analysis of interscapular SV cells and adipocytes. Stromal–vascular (SV) cells and adipocytes were fractionated by centrifugation after collagenase digestion of the adipose tissues of 10-day-old hamsters. mRNA-seq analysis was performed to identify differentially expressed genes in the SV cells from interscapular (Int) adipose tissues and adipocytes (Adip) from both the interscapular and inguinal (Ing) adipose tissues **(A,B)**. Genes encoding proteins classified as “cytokines” or “growth factors” were extracted, and the number of genes highly expressed in each cell type is shown in the Venn diagram **(A)** and heatmap **(B)**. In heatmap, the log values of fold changes compared to inguinal adipocytes (the Int-SV and the Int-SV/Int-Adipo groups) or interscapular SV cells (the Int-Adip/Ing-Adip and the Ing-Adipo groups) are shown. **(C)** The expression of genes encoding proteins belonging to the BMP family in Int-SV group were analyzed by real-time PCR. The expression of each gene was normalized to the expression of *Actb* and expressed relative to the value of the adipocyte fraction. Values represent means ± standard error for four independent experiments. Asterisks indicate significant difference (*p* < 0.05) between groups. The mean Ct values in the SV cells are shown in parentheses. **(D)** Gene enrichment analysis was done using a set of genes that was highly expressed in SV cells compared with adipocytes. Significantly enriched pathways with a GO term of “signaling” were extracted.

To explore the inhibitor and promoter of brown adipogenesis secreted from adipocytes and SV cells, respectively, mRNA-seq analysis was conducted, and gene expression was compared among three types of cells: interscapular SV cells, interscapular adipocytes, and inguinal adipocytes. Genes classified as “cytokines” or “growth factors” that exhibited an expression value (TMM-normalized counts) differing by more than 2-fold between groups were selected. As shown in Figure 5A, 19 genes were found to be highly expressed in interscapular SV cells alone (Int-SV group), whereas no genes were identified that were highly expressed in the interscapular adipocytes alone (Int-Adip group). Int-SV group contained genes for bone morphogenetic protein (BMP) family members BMP3b, BMP5, and BMP7, which are involved in BAT formation in mice (Figure 5B; Blazquez-Medela et al., 2019). These results were validated by real-time PCR (Figure 5C). Genes for vascular endothelial growth factor (VEGF) -D and nerve growth factor, which promote angiogenesis and neurogenesis and are deeply related to BAT function (Xue et al., 2009; Wang and Seale, 2016), were also found in the Int-SV group. The group of genes highly expressed in interscapular SV cells and adipocytes compared with inguinal adipocytes (Int-SV/Int-Adip group) included *Gdf15* and *Nrg4*, which is also known as BATkine (Wang et al., 2014; Campderros et al., 2019). In contrast, adipokine *Lep* was highly expressed in adipocytes irrespective of depots compared with interscapular SV cells (Int-Adip/Ing-Adip group).

To identify the activated signaling pathway in interscapular SV cells, a set of 351 genes that were more than 5-fold higher in expression in interscapular SV cells compared with adipocytes was used for enrichment analysis. Signaling pathways related to MAPK [MAPK cascade, –log10(P) = 4.0; ERK1 and ERK2 cascade, –log10(P) = 2.4] and BMP [BMP signaling pathway, –log10(P) = 3.7; transforming growth factor-β (TGF-β) receptor signaling pathway, –log10(P) = 3.6; SMAD protein signal transduction, –log10(P) = 2.5] were enriched (Figure 5D). These results suggest that BMP family proteins secreted from SV cells activate progenitors to induce differentiation into brown adipocytes.

**FIGURE 6:**
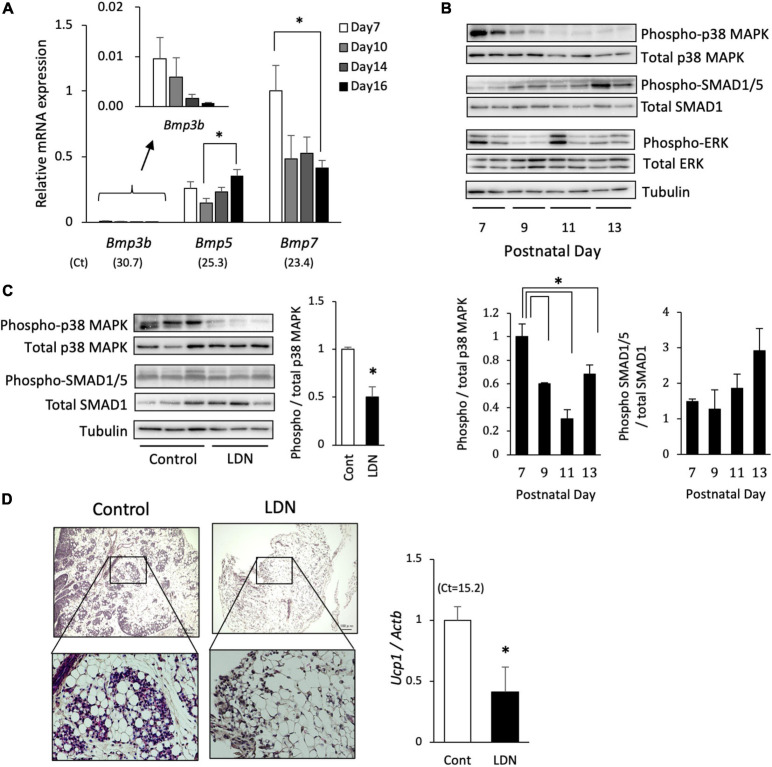
Effect of BMP signaling inhibition on postnatal development of brown adipose tissue in hamsters. **(A)** The mRNA expression of BMPs in interscapular adipose tissue of 7- to 16-day-old hamsters were analyzed by real-time PCR. The expression of each gene was normalized to the expression of *Actb* and expressed as mean values ± standard error for four independent experiments. Asterisks indicate significant differences (*p* < 0.05) between groups. The mean Ct values in Day 7 are shown in parentheses. **(B)** Phosphorylation levels of p38 mitogen-activated protein kinase (MAPK), SMAD1/5, and ERK in interscapular adipose tissue of 7- to 13-day-old Syrian hamsters were analyzed by Western blotting. Representative images are shown. Graphs show mean values ± standard error for four independent experiments. Asterisks indicate significant differences (*p* < 0.05) between groups. **(C)** LDN-193189 (LDN; 3 mg/kg), an inhibitor of type I BMP receptor, was injected to 10-day-old hamsters and interscapular adipose tissue was collected 30 min later. Phosphorylation levels of p38 MAPK and SMAD1/5 were analyzed by Western blotting. Graph shows mean values ± standard error for four independent experiments. Asterisk indicate significant differences (*p* < 0.05) compared to the control group. **(D)** LDN (3 mg/kg) was injected daily to 5-day-old hamsters for 5 days. Histological images of interscapular adipose tissues of 10-day-old hamsters show area of clusters of progenitors surrounded by white adipocytes in the control group, whereas the tissue was primarily composed of white adipocytes and small number of progenitors were observed at the edge of the tissue in the LDN-treated group. The expression of *Ucp1* was measured and normalized to the expression of *Actb* and expressed relative to the value of the control group. Values represent means ± standard error for six and eight samples for the control and the LDN group, respectively. Asterisk indicate significant differences (*p* < 0.05) compared to the control group.

The role of BMP signaling during the postnatal BAT formation was examined *in vivo*. The mRNA expression level of *Bmp3b* in interscapular adipose tissue was very low compared with those of *Bmp5* and *Bmp7* (Figure 6A). The *Bmp7* expression tended to be high during early stage and decreased thereafter. The phosphorylation of p38 MAPK was also high during early stage and gradually decreased thereafter, whereas that of SMAD1/5 failed to show a significant change during postnatal development (Figure 6B). Postnatal day-dependent change was not observed in the phosphorylation of ERK. The administration of LDN (3 mg/kg), an inhibitor of type I BMP receptor activin receptor-like kinase (ALK)-2/3, to 10-day-old hamsters significantly reduced the level of phosphorylated p38 MAPK but not that of SMAD1/5 (Figure 6C), suggesting that p38 MAPK pathway was primarily activated by BMPs at this time point. To examine the role of BMP-induced activation of p38 MAPK pathway during BAT formation process, LDN (3 mg/kg) was injected daily to 5-day-old hamsters for 5 days. At 10 days of age, interscapular adipose tissues of control hamsters consisted of brown adipocyte progenitors and white adipocytes (Figure 6D). In contrast, interscapular adipose tissues of LDN-treated hamsters were filled with white adipocytes and small number of progenitors were observed at the edge of the tissue. *Ucp1* mRNA expression was significantly reduced by the LDN treatment, indicating that BMPs – ALK 2/3 – p38MAPK pathway is critical for the BAT formation in hamsters.

## Discussion

In hamsters, BAT postnatally develops by conversion from WAT. During this process, progenitors proliferate to increase their number to replace whole tissue, simultaneously with white adipocyte disappearance, and then differentiate into brown adipocytes (Okamatsu-Ogura et al., 2018). In this study, we investigated the role of the interaction between progenitors and adipocytes in the BAT formation process. First, we established a primary culture of SV cells isolated from interscapular adipose tissue from 10-day-old pups, which consisted of progenitors and white adipocytes. Interestingly, SV cells spontaneously differentiated into brown adipocytes expressing the brown adipocyte marker, *Ucp1*. Generally, adipose tissue-derived SV cells exhibit a fibroblast-like morphology *in vitro*, and treatment with adipogenic induction cocktail such as dexamethasone and insulin are required for adipogenesis. Indeed, spontaneous differentiation was not observed in SV cells from adult hamsters where BAT formation had already been completed. Therefore, the spontaneous differentiation of SV cells from developing hamsters indicates that the progenitors have a high potential for differentiation compared with those from adult hamsters.

It is also possible that differentiation of the progenitors was stimulated by another mechanism. Consistent with this, the interscapular SV cell-conditioned medium increased expression of *Ucp1* in a dose-dependent manner, suggesting that SV cells secrete some factor(s) that induces the differentiation of progenitors in an autocrine manner. Interestingly, the interscapular SV cell-conditioned medium did not affect the expression of marker genes of adipogenesis or mature adipocytes. It is possible that factors secreted from SV cells enhances brown adipogenic program, but not adipogenesis itself, in progenitors. Since SV cells include several types of cells in addition to progenitors, such as endothelial or immune cells (Machida et al., 2018), it is unclear which type of cell secretes a stimulating factor for brown adipocyte differentiation. It has been reported that paracrine and endocrine factors, such as IGF-1 (Boucher et al., 2016), FGF-1 (Jonker et al., 2012), and FGF-10 (Sakaue et al., 2002), control adipose tissue development. Macrophages are also reported to be involved in beige adipocyte differentiation (Fischer et al., 2017; Machida et al., 2018), although the mechanism is controversial (Fischer et al., 2017; Boulet et al., 2020). Endothelial cells secrete platelet-derived growth factor (PDGF) that induces differentiation of beige adipocyte progenitors by binding to its receptor, PDGFRα (Seki et al., 2016). Thus, it is likely that the local environment is important for regulating brown adipogenesis. Of note, the promoting effect of the conditioned medium on brown adipocyte differentiation was not observed in SV cells isolated from adult hamsters. Thus, SV cells secrete promoting factors; however, the high adipogenic potential of progenitors is also indispensable for their action. Otherwise, the expression of receptors for these factors would change depending on growth.

To explore the factors secreted from SV cells, mRNA-seq analysis was conducted. Nineteen genes were highly expressed in interscapular SV cells compared with interscapular or inguinal adipocytes. Of these, three genes encoding BMP family proteins, BMP3b, BMP5, and BMP7, were identified. BMPs belong to the TGFβ family and are known to be involved in adipose tissue development and adipogenesis (Blazquez-Medela et al., 2019) by activating intracellular signaling pathways, such as SMAD and MAPK (Jia and Meng, 2021). BMPs have been classified into several subgroups, BMP2/4, BMP5/6/7/8, BMP9/10, and BMP12/13/14, and show distinct effects on adipocytes (Blazquez-Medela et al., 2019): BMP2 and BMP4 are reported to be involved in white adipogenesis (Guiu-Jurado et al., 2016; Modica et al., 2016; Denton et al., 2019), whereas BMP6, 7, and 8B are associated with brown adipogenesis. For example, BMP6 induces brown adipogenesis from skeletal muscle precursor cells (Sharma et al., 2014), whereas BMP8b is secreted from brown adipocytes and promotes thermogenesis through autocrine effects on brown adipocytes (Whittle et al., 2012). BMP7 treatment induces the commitment of mesenchymal stem cells to the brown adipogenic lineage (Tseng et al., 2008), and the deletion of the *Bmp7* gene in mice resulted in defective BAT formation (Tseng et al., 2008). The role of BMP5 and BMP3b in brown adipogenesis is unknown so far, although it was reported that BMP3b is highly expressed in preadipocytes and suppresses adipogenesis in 3T3-L1 pre-white adipocytes (Hino et al., 2012). In this study, enrichment analysis of highly expressed genes in SV cells revealed that the BMP and MAPK pathways, both major signaling pathways of BMPs, were activated in interscapular SV cells, and phosphorylation of SMAD 1/5 and p38 MAPK was detected in interscapular adipose tissue during BAT formation. Furthermore, treatment of developing hamsters with LDN, which inhibits BMP type 1 receptors ALK2 and 3, caused a significant reduction of p38 MAPK phosphorylation and drastically suppressed the BAT formation. Collectively, these results indicate that interscapular SV cells of developing hamsters secrete BMPs to induce differentiation or enhance commitment of brown adipocyte progenitors.

In contrast to the role of SV cells, spontaneous differentiation of progenitors was suppressed by co-culture with white adipocytes. Adipocytes share cytoplasm and signals through gap junctions (Zhu et al., 2016); however, a direct interaction between adipocytes and progenitors is not required because the inhibition of spontaneous differentiation was also observed after the addition of adipocyte-conditioned medium. These results suggest that interscapular adipocytes, but not inguinal adipocytes, from developing hamsters secrete some factor(s) that suppress the differentiation of the progenitors. However, no candidate was identified in the RNA-seq analysis with criteria for highly expressed genes in interscapular adipocytes compared with both interscapular SV cells and inguinal adipocytes. Recently, it has been reported that adipocytes secrete not only peptides, but also lipid molecules called “lipokines” (Li et al., 2020) or metabolites called “metabokines” (Whitehead et al., 2021). In addition, adipose tissues secrete vesicles that contain and deliver miRNA or enzymes to other cells or tissues (Thomou et al., 2017; Yoshida et al., 2019). Thus, it is plausible that the suppression factors secreted from adipocytes are non-protein factors, although there is another possibility that the factors are protein but cannot be identified by transcriptome analysis because the regulation of translation or secretion of the protein is different between interscapular adipocytes and SV cells or inguinal adipocytes in spite of the similar mRNA level. The spontaneous differentiation of progenitors *in vitro* that we observed in this study may result from their separation from surrounding white adipocytes, which inhibit differentiation. Of note, the *Ucp1* expression levels in the interscapular adipocytes from 10-day-old hamsters were as low as that in SV cells, indicating that interscapular adipocytes at this age were white rather than brown adipocytes.

A limitation to this study is that we used SV cells that contain many cell types. Therefore, it is unknown which types of cells secrete the factors that regulate adipogenesis and exhibits activated BMP and MAPK pathways. Indeed, the factors highly expressed in SV cells include VEGF-D and nerve growth factor, which induce proliferation and differentiation of endothelial cells and neurons (Chaldakov et al., 2009; Herold and Kalucka, 2020). BAT is highly vascularized and innervated with sympathetic nerves, both of which are indispensable to the thermogenic function of BAT (Xue et al., 2009; Blaszkiewicz et al., 2019). The BMP pathway plays an important role in the adult vascular endothelium and promotes angiogenesis (Dyer et al., 2014), and BMP8b secreted from brown adipocytes enhances sympathetic innervation and vascularization (Dyer et al., 2014) in addition to its effects on brown adipocytes (Pellegrinelli et al., 2018). Indeed, BAT formation in hamsters is also accompanied with sympathetic innervation and vascularization (Okamatsu-Ogura et al., 2018; Nagaya et al., 2019). Further experiments are required, for example, the purification of the progenitors using a cell surface marker protein, such as monocarboxylate transporter 1 (Iwanaga et al., 2009; Okamatsu-Ogura et al., 2018).

In this study, we found that the differentiation of brown adipocyte progenitors is positively and negatively regulated by the factors secreted from SV cells and adipocytes, respectively. It is possible that progenitors proliferate within the area of their clusters because surrounding adipocytes inhibit their differentiation. Disappearance of surrounding adipocytes leads to the release from their inhibition and may proceeds the progenitor differentiation into brown adipocyte. Although the mechanism of adipocyte disappearance is unknown, we previously showed that there was no evidence for apoptosis, and direct conversion of adipocytes from white to brown was indicated (Okamatsu-Ogura et al., 2018). In either case, this study suggests that adipocyte disappearance is a crucial event for initiating progenitor differentiation. In addition, an increase in the number of progenitors by proliferation may also enhance their differentiation through secretory factors. Collectively, this study suggests the important role of local cell–cell interactions through secretory factors and changes in cell population during BAT formation in Syrian hamsters. It is unclear if such cell–cell interactions also contribute to BAT development in other mammals. In mice, white adipose tissue develops after birth; however, adipocytes containing large unilocular lipid droplets were observed in the BAT at birth (Iwanaga et al., 2009). Beige adipogenesis may be more susceptible to the factors secreted by surrounding white adipocytes. In the past decade, the rediscovery of metabolically active BAT using radionuclide imaging techniques in adult humans has dramatically accelerated translational studies of BAT in health and disease (Saito et al., 2020). Since human BAT is a mixture of classical brown and beige adipocytes (Wu et al., 2012; de Jong et al., 2019) and surrounded by white adipocytes in most cases (Saito et al., 2009; Zingaretti et al., 2009), the regulation of adipogenesis through cell–cell interaction may be important. Further studies are required to understand adipose tissue biology, and hamsters may represent a unique model for such investigations.

## Data Availability Statement

The datasets presented in this study can be found in online repositories. The names of the repository/repositories and accession number(s) can be found below: DDBJ DRA repository accession: DRA011858 Available at: https://ddbj.nig.ac.jp/DRASearch/submission?acc=DRA011858.

## Ethics Statement

The animal study was reviewed and approved by the Animal Care and Use Committee of Hokkaido University.

## Author Contributions

JM, KN, YO-O, AT, SM, and JN-K conducted the experiments. YO-O and KK designed the experiments. JM, KN, and YO-O wrote the manuscript. All authors contributed to the article and approved the submitted version.

## Conflict of Interest

The authors declare that the research was conducted in the absence of any commercial or financial relationships that could be construed as a potential conflict of interest.

## Footnotes

1 http://metascape.org/

## Abbreviations

BAT: brown adipose tissue
BMP: bone morphogenetic protein
FGF: fibroblast growth factor
LDN: LDN-193189
PDGF: platelet-derived growth factor
SV: stromal–vascular
UCP1: uncoupling protein 1
VEGF: vascular endothelial growth factor
WAT: white adipose tissue.
